# Bridging digital and electromagnetic realms for enhanced wireless communication: current space

**DOI:** 10.1093/nsr/nwae041

**Published:** 2024-02-06

**Authors:** Qammer H Abbasi

**Affiliations:** James Watt School of Engineering, University of Glasgow, UK

## Abstract

Recently, Tie Jun Cui and team members introduced innovative macroscopic and statistical models for digital coding metasurfaces, bridging the digital and electromagnetic realms and quantifying information loss for enhanced wireless communication system design. This is a highlight of it.

Digital, coding, and programmable metasurfaces have attracted widespread attention in wireless communication fields owing to their distinguished capability in manipulating electromagnetic (EM) waves [1]. Metasurfaces consist of planar subwavelength meta-atoms and are a two-dimensional form of metamaterials [2]. These engineered surfaces can flexibly control the properties of EM waves, such as magnitude, phase, and polarization. In particular, coding metasurfaces provide a bridge between the digital world and the physical world. Accordingly, modeling and analyzing the coding metasurfaces from the perspective of information theory has become one of the most important research focuses of the scientific community [3,4].

In 2023, Tie Jun Cui and team members Rui Wen Shao, Jun Wei Wu, Zheng Xing Wang, Hui Xu, Han Qing Yang and Qiang Cheng from Southeast University introduced innovative macroscopic and statistical models for digital coding metasurfaces, bridging the digital and electromagnetic realms and quantifying information loss for enhanced wireless communication system design. In this work, the researchers proposed the concept of the *current space* for the first time, which represents the distribution and characteristics of electromagnetic currents on a metasurface. Through microwave network analysis and mathematical deductions, the researchers have obtained a concise and accurate expression that describes the mapping from the code space to the current space. In the expression, the second-order terms of the coding states are innovatively introduced to characterize mutual coupling. As an important application, the macroscopic model predicts the scattered fields of the meta-atom as accurately as full-wave simulations at lower cost. The current patterns of a representative 1-bit meta-atom and the predicted results of the macroscopic model are shown in Fig. 1. Additionally, measured results obtained from a 6 × 6 meta-elements prototype verifies the efficacy of the proposed macroscopic model.

**Figure 1.**
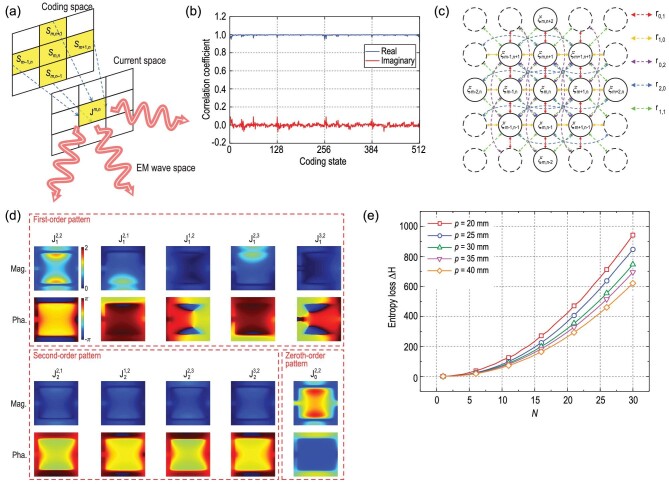
(a) Schematic diagram of the mapping from the coding space to the current space. The current on the central element depends on the coding states of the adjacent elements. (b) The 10 equivalent current patterns of the central element. They are extracted by the macroscopic model. (c) The correlation coefficients between the predicted and simulated equivalent currents. (d) Schematic diagram of the proposed statistical model for the currents on a metasurface. The circles indicate the element currents and the arrows indicate the correlation between two currents. (e) The entropy loss of metasurfaces with different periods and scales. Adapted from [5].

More importantly, the macroscopic model lays a solid foundation for analyzing the EM information of digital coding metasurfaces. The researchers also proposed a novel model that further describes the digital coding metasurfaces from a statistical perspective. Mutual coupling can introduce correlations and dependencies among elements, influencing the information content in the current space. In the model, the mutual coupling is transformed into correlation and the joint probability distribution function of the currents is obtained. Finally, the concept of *current entropy* is introduced to characterize the capability of metasurfaces in transforming digital information into physical information. By understanding how information is transformed and potentially lost during the coding-to-current conversion, researchers can make informed decisions about system design and optimization.

The proposed models fill up the gap between digital information and EM physics, which can help to achieve numerous practical applications, such as supporting the reconfigurable intelligence surfaces (RISs) in achieving accurate beamforming and channel state information and assisting the design of precoding codebooks in metasurface-based direct digital modulation systems to approach the upper bound of channel capacity.
